# Supplementary material for: Deep-subwavelength control of acoustic waves in an ultra-compact metasurface lens
Source: Nat Commun. 2018 Nov 22;9:4920. doi: 10.1038/s41467-018-07315-6 (PMC6250707; doi:10.1038/s41467-018-07315-6)
Supplement: Supplementary file 1 — Supplementary Information [file 41467_2018_7315_MOESM1_ESM.pdf]

Supplementary Information

Deep-subwavelength control of acoustic waves in an ultra-compact metasurface lens

Chen et al.

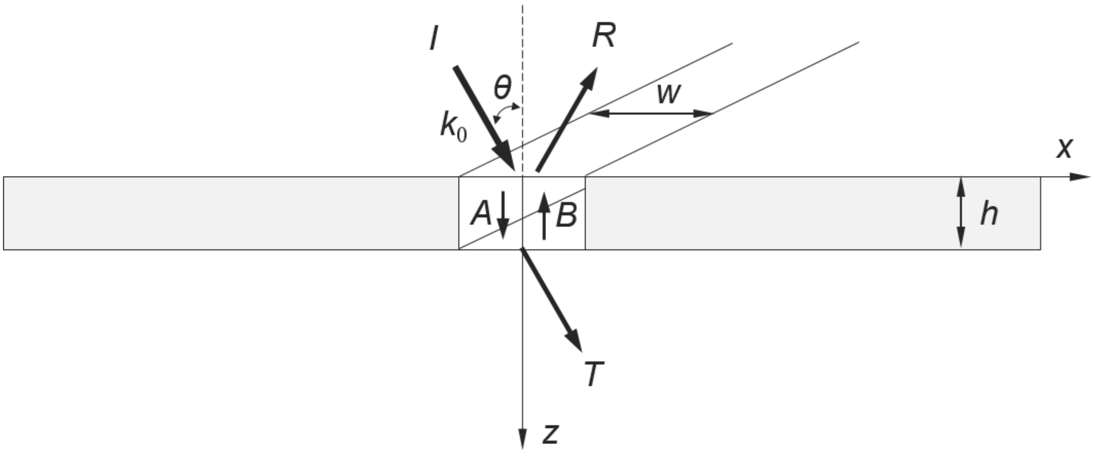

Supplementary Figure 1 | Schematic diagram of wave interactions with a subwavelength slit. The slit has a rectangular cross-section with width w and thickness h. The pressure field of incident waves is normalized to 1 Pa.

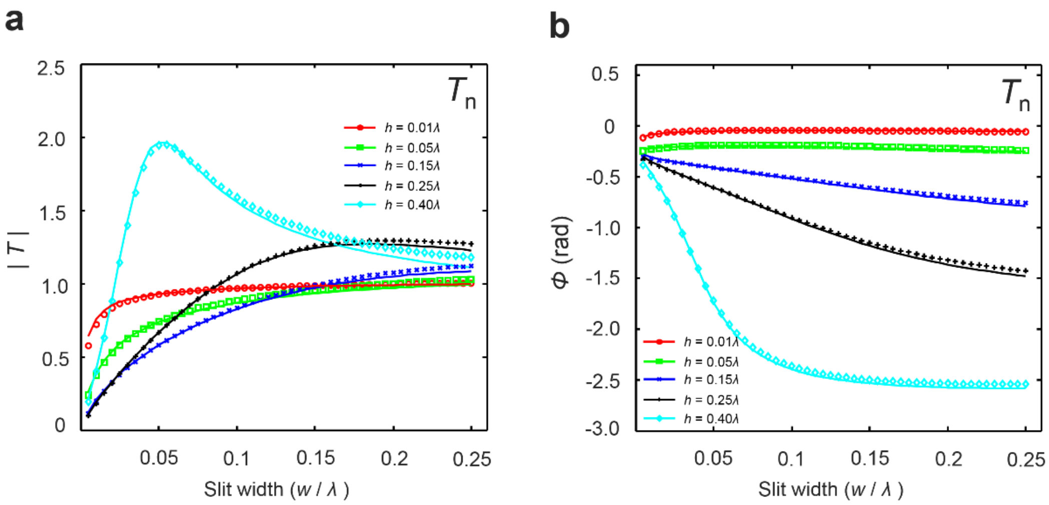

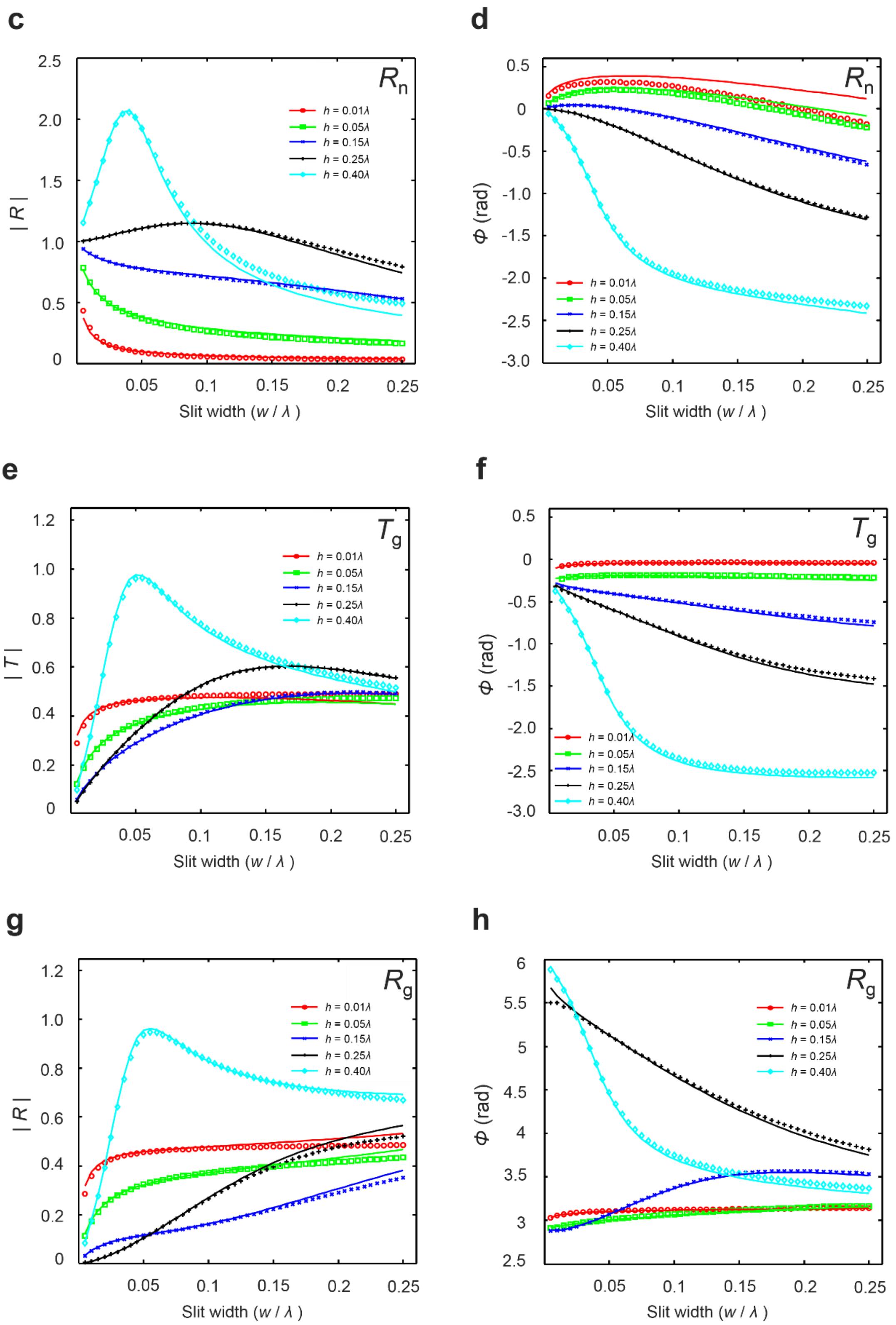

Supplementary Figure 2 | The comparisons of transmission and reflection coefficients of a single slit between theoretical calculations (solid lines) and numerical simulations (marks). The calculation and simulation for **(a, b)** T_n, **(c, d)** R_n, **(e, f)** T_g and **(g, h)** R_g are carried out on different slit parameters. The slit width increases from 0.005λ to 0.25λ with a step of 0.005λ at different thicknesses (h = 0.01λ, 0.05λ, 0.15λ, 0.25λ, 0.4λ).

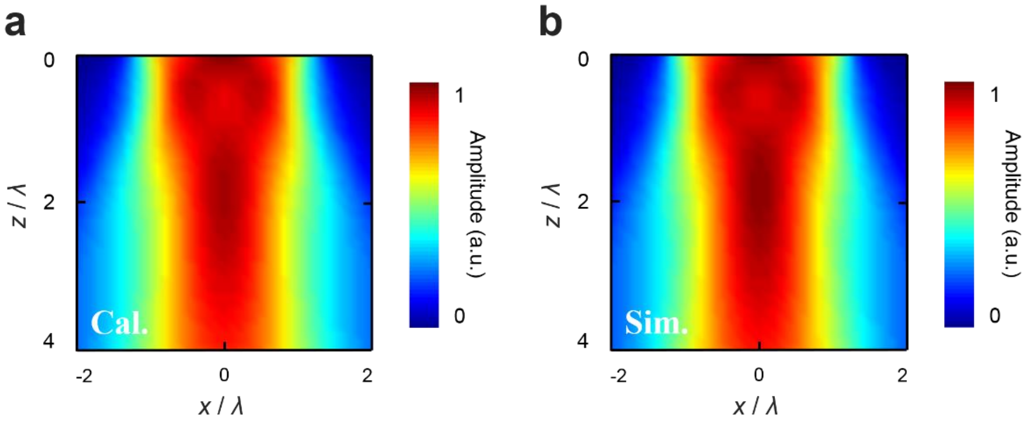

Supplementary Figure 3 | (a) Calculated and **(b)** simulated pressure field after a plane wave transmits through a user-defined acoustic metasurface. The thickness, spacing, and slit number in the acoustic metasurface are 0.1λ, 0.15λ and 19, respectively. The slit widths decrease from 0.1λ to 0.01λ with a step of 0.01λ from the center to the side.

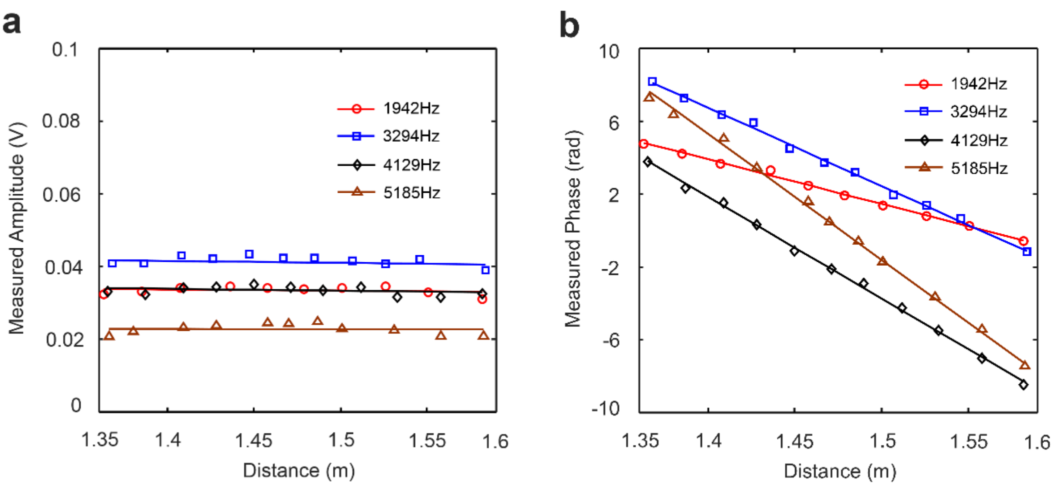

Supplementary Figure 4 | (a) The amplitude and **(b)** phase of the acoustic signals generated by the plane wave speaker at selected frequencies: 1942Hz (circles), 3294Hz (squares), 4129Hz (diamonds) and 5185Hz (triangles). The measurements were performed along the 45° diagonal path in front of the speaker on which the microphone slides. The distance of the measurement locations was recorded with respect to a reference point on the frame diagonal. The measured phase data was fitted using linear least squares, and the parameters are listed in Supplementary table 3.

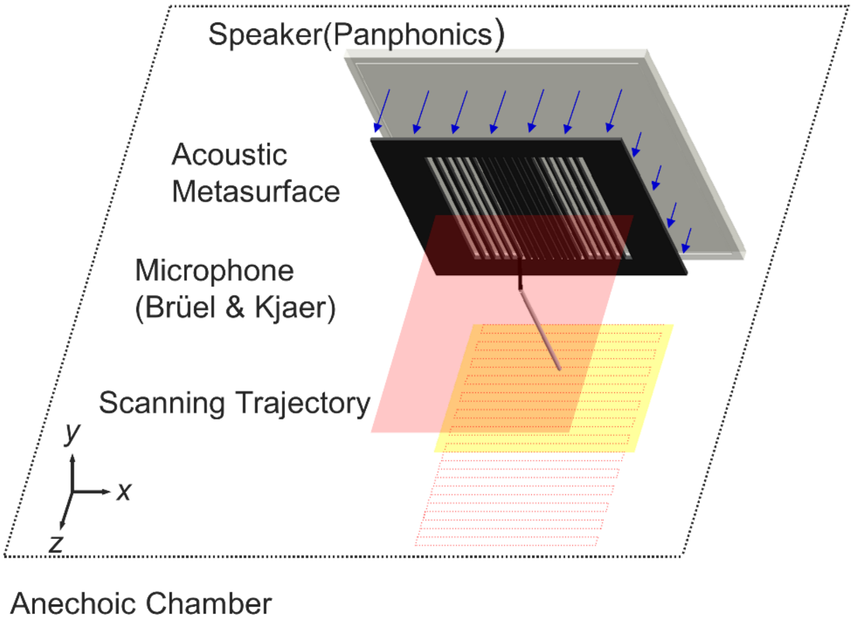

Supplementary Figure 5 | Schematic of the experimental setup for acoustic field measurements.

m	1	2	3	4	5	6	7	8	9	10
slit width (0.01λ)	**14.1**	**12.3**	**12.6**	**12.6**	**11.9**	**7.0**	**3.0**	**2.0**	**2.0**	**2.1**
m	11	12	13	14	15	16	17	18	19	
slit width (0.01λ)	**2.0**	**2.0**	**3.0**	**7.0**	**11.9**	**12.6**	**12.6**	**12.3**	**14.1**	

Supplementary Table 1: Optimized slit widths of the acoustic metasurface lens for far-field sound focusing.

m	1	2	3	4	5	6	7	8	9	10	11
slit width (0.01λ)	**0.3**	**0.32**	**0.34**	**5.32**	**1.58**	**8.94**	**1.58**	**5.32**	**0.34**	**0.32**	**0.3**

Supplementary Table 2: Optimized slit widths of the near-field metasurface lens.

Frequency (Hz)	Theoretical phase slope (rad m^-1)	Experimental phase slope (rad m^-1)	R^2 value
1942	-25.15	-24.47	0.9954
3294	-42.67	-43.13	0.9981
4129	-53.48	-56.02	0.9986
5185	-67.16	-67.06	0.9969

Supplementary Table 3: The linear least square fitting parameters of the measured phases.

Supplementary Note 1. The Derivation of transmission and reflection coefficients

Consider an amplitude- normalized plane wave with a tangential component of wave-vector α_0 incident on a rigid plate, as shown in Supplementary Fig. 1. The slit has a rectangular cross-section with width *w* and thickness *h*. Under this insonfication, the expression of the pressure field above and below the slit can be, respectively, written as

$$P_{\mathrm{a}} = P_{\mathrm{I}} + P_{\mathrm{r}} = \mathrm{e}^{i\alpha_0 x + i\eta_0 z} + \int_{-\infty}^{+\infty} R(\alpha)\mathrm{e}^{i\alpha x - i\eta z} d\alpha \tag{A.1}$$

and

$$P_{\mathrm{b}} = P_{\mathrm{t}} = \int_{-\infty}^{+\infty} T(\alpha)\mathrm{e}^{i\alpha x + i\eta(z-h)} d\alpha , \tag{A.2}$$

where $i = \sqrt{-1}$, $\eta_0 = \sqrt{k_0^2 - \alpha_0^2}$ is the z component of the incident-wave vector, α and $\eta = \sqrt{k_0^2 - \alpha^2}$ are the wave vectors of high-order diffraction along *x* and *z* directions, respectively; $k_0 = \omega / c$ is the wave-vector of the incident wave in air (ω is the angular frequency and *c* is the acoustic velocity in air); $R(\alpha)$ and $T(\alpha)$ are the reflection and transmission coefficients, respectively. By making Fourier transforms of each waveguide modes, $R(\alpha)$ and $T(\alpha)$ can be expanded as[1]

$$R(\alpha) = \delta(\alpha - \alpha_0) + \sum_{j\geq 0} R_j W_j(\alpha)/\eta \tag{A.3}$$

$$T(\alpha) = \sum_{j\geq 0} T_j W_j(\alpha)/\eta , \tag{A.4}$$

where δ is the Dirac delta function, and

$$W_j(\alpha) = \int_{-w/2}^{w/2} \mathrm{e}^{-i\alpha x} \cos(\frac{j\pi}{w} x) dx . \tag{A.5}$$

Inside the slit, the pressure field is also expanded in terms of the waveguide modes, which is expressed as

$$P_{\mathrm{in}} = \sum_{j\geq 0} \cos(\frac{j\pi}{w} x)[A_j \,\mathrm{e}^{i\sqrt{k_0^2 - (\frac{j\pi}{w})^2} z} + B_j \mathrm{e}^{-i\sqrt{k_0^2 - (\frac{j\pi}{w})^2} z}] \tag{A.6}$$

where A_j and B_j are the pressure amplitudes of the forward and backward waves of *j*-th waveguide mode, and *j* is a positive integer.

In the case of a subwavelength slit, the pressure field can be sufficiently well described by the first term in the mode expansions (this means that only the fundamental mode (j = 0) is retained)[1]. Therefore, Supplementary equations 1-6 can be simplified to

$$\begin{cases} P_{\mathrm{a}} = \mathrm{e}^{i\alpha_0 x + i\eta_0 z} + \mathrm{e}^{i\alpha_0 x - i\eta_0 z} + \int_{-\infty}^{+\infty} \frac{R_0 W_0(\alpha)}{\eta} \mathrm{e}^{i\alpha x - i\eta z}\, d\alpha \\ P_{\mathrm{in}} = A_0\, \mathrm{e}^{ik_0 z} + B_0\, \mathrm{e}^{-ik_0 z} \\ P_{\mathrm{b}} = \int_{-\infty}^{+\infty} \frac{T_0 W_0(\alpha)}{\eta} \mathrm{e}^{i\alpha x + i\eta(z-h)}\, d\alpha \end{cases} \quad \text{(A.7-1)}$$

with $W_0(\alpha) = \int_{-w/2}^{w/2} \mathrm{e}^{-i\alpha x}\, dx = \frac{2\sin(w\alpha/2)}{\alpha}$.

The normal velocities can then be obtained from $u = \frac{1}{i\rho\omega}\frac{\partial p}{\partial z}$, and thus we have

$$\begin{cases} u_{\mathrm{a}} = \frac{1}{\rho\omega}(\eta_0\, \mathrm{e}^{i\alpha_0 x + i\eta_0 z} - \eta_0\, \mathrm{e}^{i\alpha_0 x - i\eta_0 z} - \int_{-\infty}^{+\infty} R_0 W_0(\alpha) \mathrm{e}^{i\alpha x - i\eta z}\, d\alpha) \\ u_{\mathrm{in}} = \frac{k_0}{\rho\omega}(A_0\, \mathrm{e}^{ik_0 z} - B_0\, \mathrm{e}^{-ik_0 z}) \\ u_{\mathrm{b}} = \frac{1}{\rho\omega}(\int_{-\infty}^{+\infty} T_0 W_0(\alpha) \mathrm{e}^{i\alpha x + i\eta(z-h)}\, d\alpha) \end{cases} \quad \text{(A.7-2)}$$

where ρ is the mass density of air. According to the boundary conditions, the pressure field and normal velocity at the interfaces (i.e., z = 0 and z = h) should be continuous. Combining the continuity conditions with Supplementary equation 7, we can determine the coefficients R_0, T_0, A_0, and B_0.

Firstly, we calculate the transmission and reflection coefficients [T_n and R_n] for a normally incident plane wave. Under the normal incidence (θ = 0), we have α_0 (=$k_0 \sin\theta$) = 0 and $\eta_0 = k_0$. By substituting the initial conditions to Supplementary equation 7 and applying the boundary conditions at z = 0, we obtain

$$\begin{cases} 2 + \int_{-\infty}^{+\infty} \frac{R_0 W_0(\alpha)}{\eta} \mathrm{e}^{i\alpha x}\, d\alpha = A_0 + B_0 \\ \int_{-\infty}^{+\infty} R_0 W_0(\alpha) \mathrm{e}^{i\alpha x}\, d\alpha = -k_0(A_0 - B_0) \end{cases} \quad \text{(A.8-1)}$$

Similarly, at $z = h$

$$\begin{cases} \int_{-\infty}^{+\infty} \frac{T_0 W_0(\alpha)}{\eta} \mathrm{e}^{i\alpha x}\, d\alpha = A_0\, \mathrm{e}^{ik_0 h} + B_0\, \mathrm{e}^{-ik_0 h} \\ \int_{-\infty}^{+\infty} T_0 W_0(\alpha) \mathrm{e}^{i\alpha x}\, d\alpha = k_0(A_0\, \mathrm{e}^{ik_0 h} - B_0\, \mathrm{e}^{-ik_0 h}) \end{cases} \quad \text{(A.8-2)}$$

Note that although the slits are deep-subwavelength compared to the incident wave, they cannot be simply considered as points for all waves, especially the evanescent waves with large tangential wave-vectors. As the integration is performed for all wave components over [-∞, ∞], here we generalize the evaluation of $\mathrm{e}^{i\alpha x}$ by averaging it on the whole slit width, to integrate out the x

dependence. Thus, we have $e^{i\alpha x} \approx \frac{1}{w}\int_{-w/2}^{w/2} e^{i\alpha x}\,dx = \frac{2\sin(w\alpha/2)}{\alpha w}$. Substituting $e^{i\alpha x}$ and $W_0(\alpha) = \frac{2\sin(w\alpha/2)}{\alpha}$ into Supplementary equation 8, we therefore have

$$\begin{cases} T_{\mathrm{n}} = \int_{-\infty}^{\infty} \frac{T_0 W_0(\alpha)}{\eta} e^{i\alpha x} d\alpha = \frac{4Q e^{ik_0 h}}{(Q+1)^2 - (Q-1)^2 e^{2ik_0 h}} \\ R_{\mathrm{n}} = 1 + \int_{-\infty}^{\infty} \frac{R_0 W_0(\alpha)}{\eta} e^{i\alpha x} d\alpha = \frac{Q-1}{Q+1}(T_{\mathrm{n}} e^{ik_0 h} - 1) \end{cases} \tag{A.9}$$

with $Q = \frac{k_0}{\pi w}\int_{-\infty}^{\infty} \frac{1-\cos(w\alpha)}{\alpha^2\sqrt{k_0^2-\alpha^2}} d\alpha$.

Similar calculations are implemented to the grazing incidence by changing the initial conditions to $\alpha_0 = k_0$ and $\eta_0 = 0$, and finally we get

$$\begin{cases} T_{\mathrm{g}} = \frac{2Q e^{ik_0 h}\mathrm{sinc}(k_0 w/2)}{(Q+1)^2 - (Q-1)^2 e^{2ik_0 h}} \\ R_{\mathrm{g}} = \frac{T_{\mathrm{g}} e^{ik_0 h}(Q-1) + \mathrm{sinc}(k_0 w/2)}{Q+1} - 1 \end{cases} \tag{A.10}$$

Supplementary Note 2. Characteristic parameters of the cylindrical waves (CWs)

Under uniform illumination by a (normal or grazing) plane wave, the acoustic wave scattered by a subwavelength slit can be regarded as a new point source that thus generates a CW. The excited CW can be characterized by two coefficients, β and ϕ. To extract the coefficients, a two-step procedure is adopted. Firstly, the pressure field is extracted through a numerical simulation. Then, using Eq. (1) in the main text, the coefficients are optimized by fitting the pressure field distribution over an interval ($w/2 \leq r \leq 10\lambda$). This procedure offers a rigorous numerical approach to calculate β and ϕ, and similar calculations are performed for different slit parameters. The slit width increases from 0.005λ to 0.2λ with a step of 0.005λ at different thicknesses (h = 0.05λ, 0.1λ, 0.3λ, 0.7λ, 0.9λ). The calculated β and ϕ for different slits are provided in the Source Data file. From these calculations, it can be found that both β and ϕ are relying on the slit width w, but independent of thickness h. To determine the relationship between the CWs and the slit parameters, β and ϕ are fitted as a polynomial function of slit width with the least-mean-square method.

Supplementary References

1. Takakura, Y. Optical resonance in a narrow slit in a thick metallic screen. *Phys. Rev. Lett*. 86, 5601 (2001).
