# Supplementary material for: Deep-subwavelength control of acoustic waves in an ultra-compact metasurface lens
Source: Nat Commun. 2018 Nov 22;9:4920. doi: 10.1038/s41467-018-07315-6 (PMC6250707; doi:10.1038/s41467-018-07315-6)
Supplement: Supplementary file 3 — Source Data file [file 41467_2018_7315_MOESM3_ESM.pdf]

The fitted β and ϕ for different slit parameters

w	$h = 0.05\lambda$		$h = 0.1\lambda$		$h = 0.3\lambda$		$h = 0.7\lambda$		$h = 0.9\lambda$	
	β	ϕ	β	ϕ	β	ϕ	β	ϕ	β	ϕ
0.005λ	0.29	-1.26	0.3	-1.26	0.3	-1.27	0.3	-1.27	0.3	-1.27
0.010λ	0.34	-1.22	0.34	-1.22	0.34	-1.23	0.34	-1.22	0.34	-1.23
0.015λ	0.37	-1.19	0.37	-1.19	0.37	-1.19	0.37	-1.19	0.37	-1.2
0.020λ	0.39	-1.16	0.39	-1.16	0.39	-1.17	0.39	-1.16	0.39	-1.17
0.025λ	0.41	-1.14	0.41	-1.14	0.41	-1.14	0.41	-1.14	0.41	-1.15
0.030λ	0.43	-1.12	0.43	-1.12	0.43	-1.12	0.43	-1.12	0.42	-1.12
0.035λ	0.45	-1.1	0.45	-1.1	0.44	-1.1	0.44	-1.1	0.44	-1.1
0.040λ	0.46	-1.08	0.46	-1.08	0.46	-1.09	0.46	-1.08	0.45	-1.07
0.045λ	0.48	-1.06	0.47	-1.06	0.47	-1.07	0.47	-1.07	0.46	-1.05
0.050λ	0.49	-1.05	0.49	-1.05	0.48	-1.05	0.49	-1.05	0.48	-1.03
0.055λ	0.5	-1.03	0.5	-1.03	0.49	-1.04	0.5	-1.04	0.49	-1.01
0.060λ	0.51	-1.02	0.51	-1.02	0.5	-1.02	0.51	-1.02	0.5	-0.99
0.065λ	0.52	-1	0.52	-1	0.51	-1	0.52	-1.01	0.52	-0.98
0.070λ	0.53	-0.99	0.53	-0.99	0.52	-0.99	0.53	-0.99	0.53	-0.96
0.075λ	0.54	-0.97	0.54	-0.98	0.53	-0.97	0.54	-0.98	0.54	-0.95
0.080λ	0.55	-0.96	0.55	-0.96	0.54	-0.96	0.55	-0.97	0.55	-0.94
0.085λ	0.56	-0.95	0.56	-0.95	0.55	-0.95	0.56	-0.95	0.56	-0.92
0.090λ	0.57	-0.94	0.57	-0.94	0.56	-0.93	0.57	-0.94	0.57	-0.91
0.095λ	0.58	-0.92	0.58	-0.93	0.57	-0.92	0.57	-0.93	0.58	-0.9
0.100λ	0.59	-0.91	0.59	-0.91	0.58	-0.9	0.58	-0.92	0.59	-0.89
0.105λ	0.6	-0.9	0.59	-0.9	0.58	-0.89	0.59	-0.9	0.6	-0.88
0.110λ	0.6	-0.89	0.6	-0.89	0.59	-0.88	0.6	-0.89	0.6	-0.86
0.115λ	0.61	-0.88	0.61	-0.88	0.6	-0.86	0.6	-0.88	0.61	-0.85
0.120λ	0.62	-0.86	0.62	-0.87	0.61	-0.85	0.61	-0.87	0.62	-0.84
0.125λ	0.63	-0.85	0.62	-0.86	0.61	-0.84	0.62	-0.86	0.63	-0.83
0.130λ	0.63	-0.84	0.63	-0.85	0.62	-0.83	0.62	-0.85	0.64	-0.82
0.135λ	0.64	-0.83	0.64	-0.83	0.63	-0.81	0.63	-0.83	0.64	-0.81
0.140λ	0.65	-0.82	0.64	-0.82	0.63	-0.8	0.64	-0.82	0.65	-0.8
0.145λ	0.65	-0.81	0.65	-0.81	0.64	-0.79	0.64	-0.81	0.66	-0.79
0.150λ	0.66	-0.8	0.66	-0.8	0.65	-0.78	0.65	-0.8	0.66	-0.78
0.155λ	0.67	-0.79	0.66	-0.79	0.65	-0.77	0.65	-0.79	0.67	-0.77
0.160λ	0.67	-0.78	0.67	-0.78	0.66	-0.76	0.66	-0.78	0.67	-0.76
0.165λ	0.68	-0.77	0.67	-0.77	0.67	-0.75	0.66	-0.77	0.68	-0.75
0.170λ	0.68	-0.76	0.68	-0.76	0.67	-0.74	0.67	-0.76	0.69	-0.74
0.175λ	0.69	-0.75	0.68	-0.75	0.68	-0.73	0.67	-0.75	0.69	-0.73
0.180λ	0.69	-0.74	0.69	-0.74	0.68	-0.72	0.68	-0.74	0.7	-0.72
0.185λ	0.7	-0.73	0.69	-0.73	0.69	-0.71	0.68	-0.72	0.7	-0.71
0.190λ	0.7	-0.72	0.7	-0.72	0.69	-0.7	0.69	-0.71	0.71	-0.7
0.195λ	0.71	-0.71	0.7	-0.71	0.7	-0.69	0.69	-0.7	0.71	-0.69
0.200λ	0.71	-0.7	0.71	-0.7	0.7	-0.68	0.7	-0.69	0.72	-0.68
